# Socioeconomic Deprivation as a Predictor of Lower Limb Amputation in Diabetic Foot Disease

**DOI:** 10.7759/cureus.44426

**Published:** 2023-08-31

**Authors:** Ahmad Bilal, Danuksha K Amarasena, Anand Pillai

**Affiliations:** 1 Orthopaedics and Traumatology, Wythenshawe Hospital, Manchester, GBR; 2 Faculty of Medicine, University of Manchester, Manchester, GBR

**Keywords:** diabetic foot disease (dfd), amputation, lsoa, deprivation, index of multiple deprivation (imd)

## Abstract

Objective

The prevalence of diabetes and its complications are on the rise worldwide. This is particularly prevalent in low- and middle-income countries. The Index of Multiple Deprivation (IMD) is a measure of relative social deprivation. This index classifies England into small subsets called lower layer super output areas (LSOAs) and then ranks these LSOAs into deciles from the most deprived to the least deprived area. We used this to analyse the relationship between deprivation and outcomes in diabetic foot disease (DFD).

Methods

We evaluated patients admitted to our multidisciplinary unit with DFD over a five-year period. Their postal codes were used to classify these patients into IMD deciles with decile 1 representing the 10% of most deprived LSOAs and decile 10 the least deprived areas in the country. We used this classification to analyse the relationship between deprivation and its influence on surgical outcomes and lower limb amputation. We specifically compared those falling in the top five against the bottom five deciles.

Results

Our cohort consisted of 70 patients with diabetes who had surgery on their diabetic foot. Of this cohort, 33 (47%) of these underwent amputation. The majority of these procedures were minor amputations conducted on the forefoot (75.6%) and there were no below knee amputations. Of those requiring an amputation, 27 patients (81.8%) had an IMD decile of 5 or below, and only six (18.2%) patients had above 5.

Conclusion

Our study shows that the likelihood of amputations related to diabetic foot disease is inversely proportional to the index of multiple deprivation.

## Introduction

Approximately 537 million adults across the world are living with diabetes [1]. Of these people suffering with diabetes, three in four live in low- and middle-income countries [1,2]. Despite there being a well-established national diabetic screening program, there is no specific screening program to identify diabetic foot disease.

Diabetic foot disease affects up to 183 million people worldwide [3]. Diabetic foot neuropathy increases the risks of foot ulcers, infection, and the eventual need for amputation. Every 30 seconds a lower limb is amputated across the world as a consequence of diabetes [2] and the risk of amputation in a person with diabetes is 25 times greater compared to people without diabetes [2]. Of these amputations relating to diabetes, 85% are preceded by a foot ulcer [4].

Foot disease in diabetes arises as a consequence of either neuropathy or ischaemia of the foot. This state can eventually progress into Charcot neuropathy or ulceration of the foot which is susceptible to infection and osteomyelitis. These factors, in isolation or combination, amongst other co-morbidities such as preexisting peripheral arterial disease can in turn result in the requirement for an amputation.

The presence of diabetic foot disease and the increased need for amputation have a significant effect on the life expectancy of a patient. Whilst amputations may be necessary in reducing the immediate risk of spreading osteomyelitis and sepsis, over time they can reduce the patients’ mobility which is a vital factor in helping to maintain good glycaemic control and prevent worsening of possibly fatal diabetic complications.

## Materials and methods

Our study looked at the index of multiple deprivation deciles (of the areas that our patients resided) to analyse the association between socioeconomic deprivation and diabetic foot amputation [5].

The Indices of Deprivation (IoD) is a database in England that follows an established methodological framework to identify a relative measure of deprivation [5]. This database collects data on these seven distinct domains of deprivation: income, employment, education, health, crime, barriers to housing and services, and living environment. These domains are combined and appropriately weighted to calculate the Index of Multiple Deprivation (IMD) [5]. The IMD then divides and ranks the country into small areas called lower layer super output areas (LSOAs), of which England is split up into 32,844, with 1 representing the most deprived to 32,844 representing the least deprived area in the country. These are then further grouped into deprivation deciles as shown in Table 1.

**Table 1:** Breakdown of LSOA into IMD deciles LSOA: Lower Layer Super Output Area IMD: Index of Multiple Deprivation

Decile	LSOAs
1 (Most Deprived Areas)	1 to 3,284
2	3,285 to 6,568
3	6,569 to 9,853
4	9,854 to 13,137
5	13,138 to 16,422
6	16,423 to 19,706
7	19,707 to 22,990
8	22,991 to 26,275
9	26,276 to 29,559
10 (Least Deprived Areas)	29,560 to 32,844

There is a well-documented association between diabetes and socioeconomic deprivation [6]. The National Health Service (NHS) is constantly working to ensure that those residing in these areas of deprivation can get access to equal-quality healthcare [7].

We assessed if there was a greater incidence of diabetic foot disease-related amputation amongst those residing in areas of greater levels of deprivation (lower IMD decile). In doing so, this could help to highlight areas that are neglected so that resources can be allocated accordingly.

**Figure 1:**
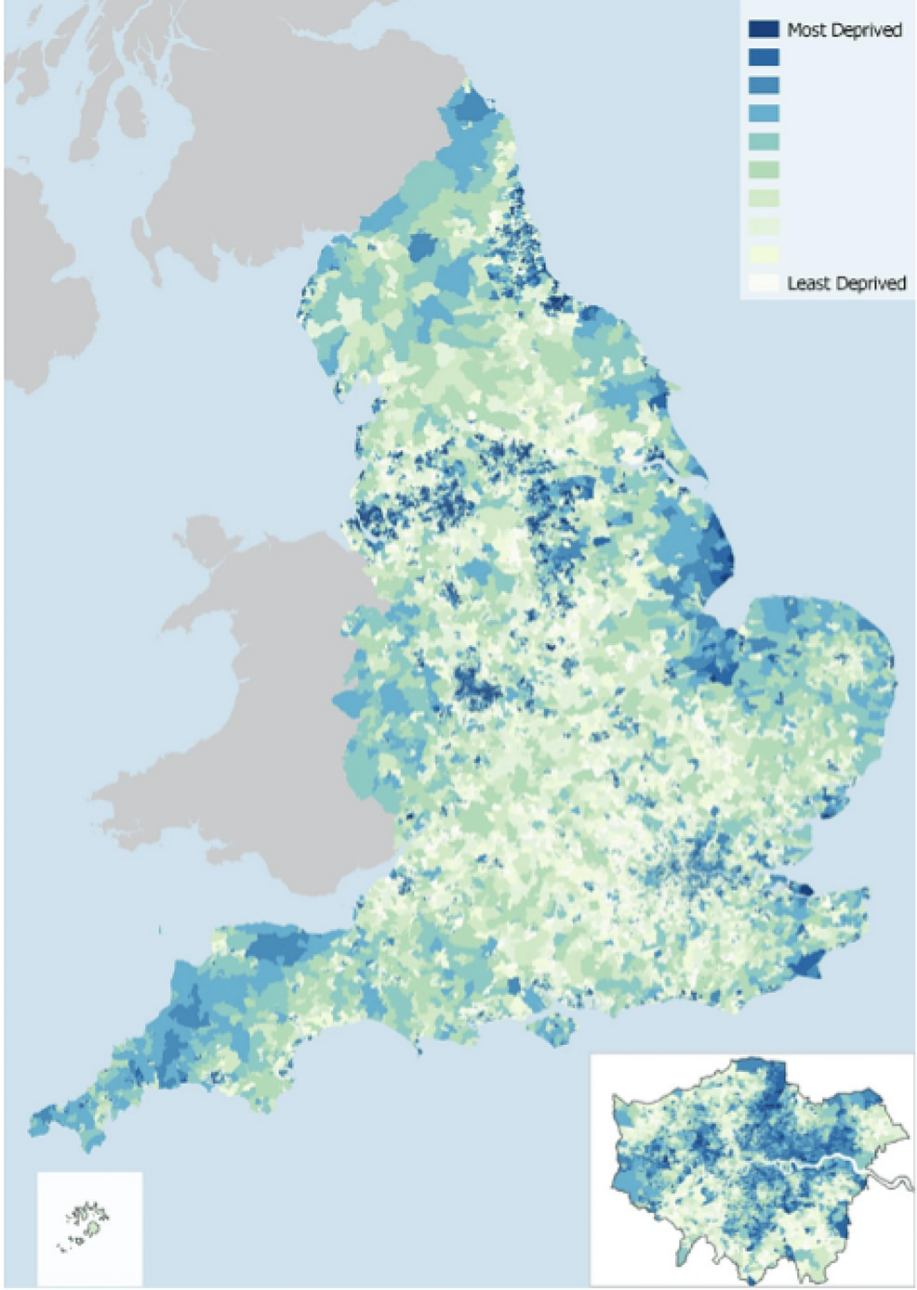
Distribution of IMD in England Map showing the distribution of Index of Multiple Deprivation scores by LSOAs in 2019 in England [5] IMD: Index of Multiple Deprivation LSOA: Lower Layer Super Output Area © Ministry of Housing, Communities & Local Government, 2019. This information is licensed under the Open Government Licence v3.0. To view this licence, visit https://www.nationalarchives.gov.uk/doc/open-government-licence/

**Figure 2:**
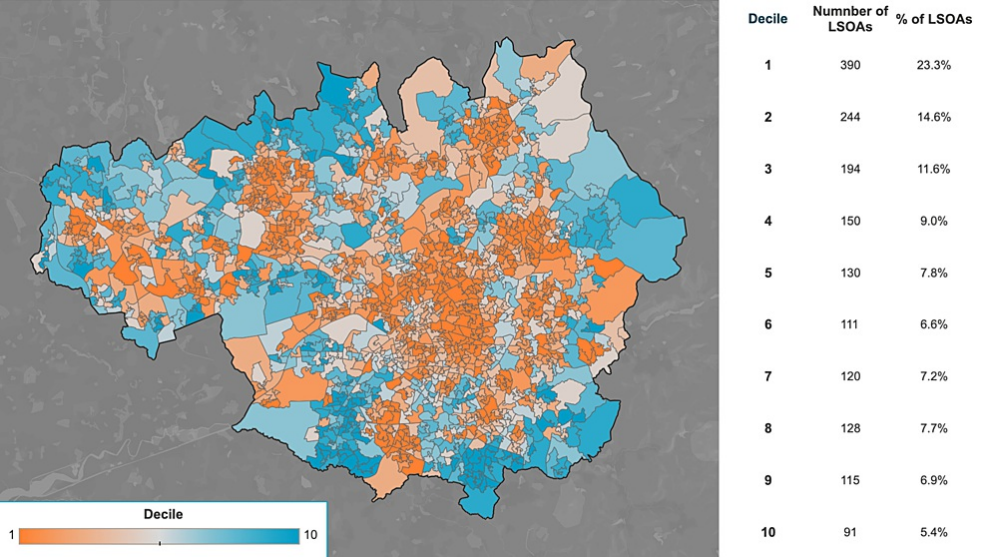
Distribution of IMD Deciles in Greater Manchester Map showing the Index of Multiple Deprivation deciles within Greater Manchester [8] IMD: Index of Multiple Deprivation Image reproduced with permission of the rights holder, Greater Manchester Poverty Action.

We retrospectively evaluated the adult patients with diabetes who were admitted to our multidisciplinary unit for a surgery relating to diabetic foot disease between March 2017 and March 2022. These procedures included: incisions and drainage, debridement, reconstruction and amputations of lower limb. For the purpose of the study, we divided these patients into two cohorts. Cohort 1 received an amputation of part of their lower limb and Cohort 2 received no amputations. We collected information regarding the postal codes of each patient and calculated the relative deprivation using the IMD (Figures 1, 2). We used this information to compare the levels of socioeconomic deprivation in each cohort and ascertain whether this could be used as an independent predictor of the need for a lower extremity amputation.

## Results

**Figure 3:**
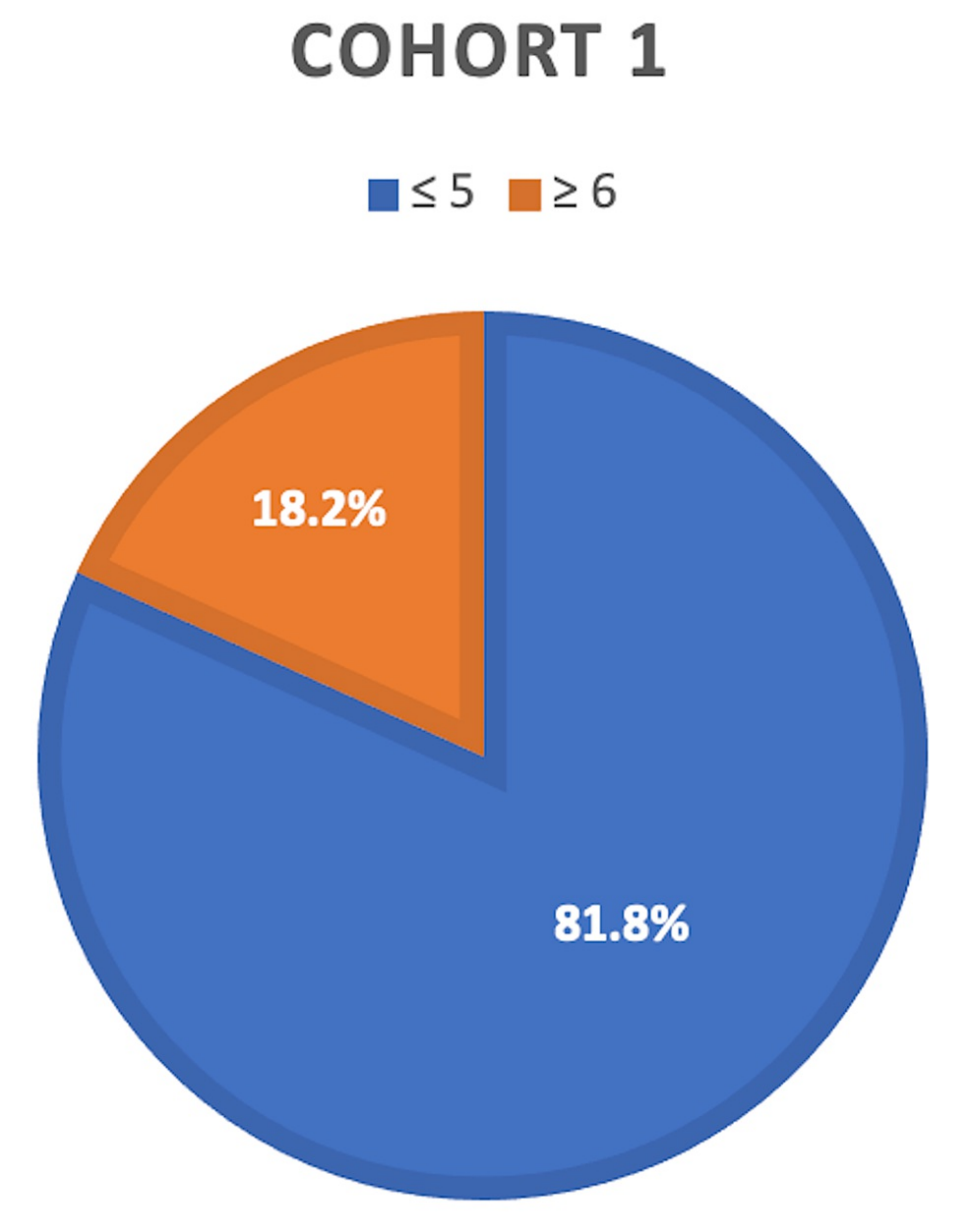
Distribution of IMD deciles amongst amputees Pie chart showing the relationship between the percentage of patients in Cohort 1 (received amputations) with IMD above and below 5 IMD: Index of Multiple Deprivation

**Figure 4:**
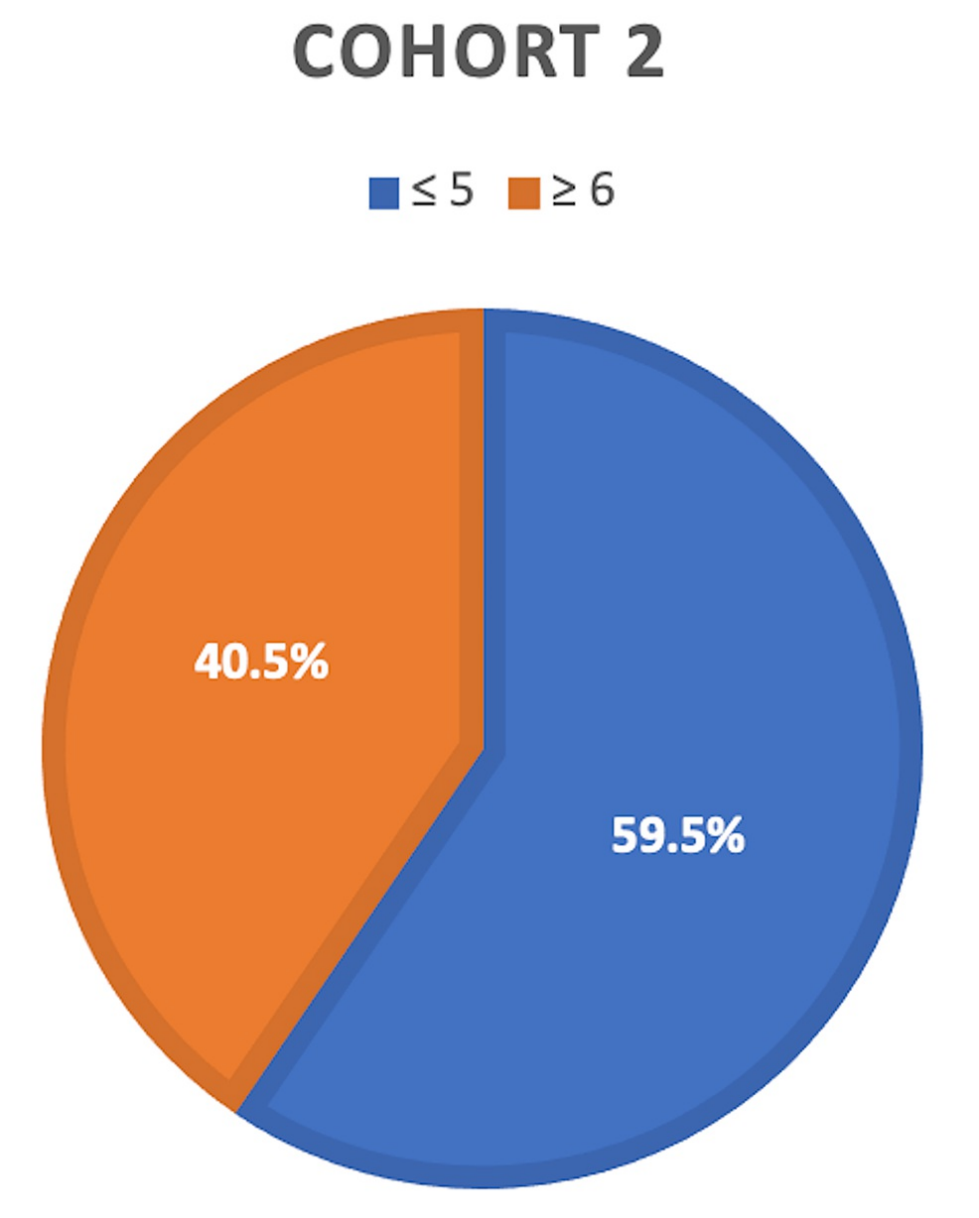
Distribution of IMD deciles amongst non-amputees Pie chart showing the relationship between the percentage of patients in Cohort 2 (received amputations) with IMD above and below 5 IMD: Index of Multiple Deprivation

There were 70 patients included in our study who had surgery for their foot disease. The majority of these were males 55 (78.6%). The mean age at diagnosis was 45.6 years (SD 15.8) and the mean age at first surgery was 61.5 years (SD 11.4). Of the 70 patients 33 (47%) required amputations. Most of these amputations were of the forefoot 75.6% followed by hindfoot (18.2%) and midfoot (6.2%). None of the patients had below-knee amputations. The mean IMD for the amputees was 9019.7 with a range of 576 to 32098. Amputations were much more common amongst those residing in areas of greater socioeconomic deprivation. This was evident as 81.2% of patients (27 patients) requiring amputations had an IMD decile of 5 or below (bottom 50% of most deprived areas in the country) compared to only 18.2% of patients (six patients) who had an IMD above 5 (Figure 3). Furthermore, a greater proportion of amputees fell within the bottom 5 deciles for deprivation compared to non-amputees (81.2% vs 59.5%), as can be seen in Figure 3 and Figure 4. Statistical analysis shows that the relative risk of amputation in patients residing in areas of low IMD (5 or less) is four times greater than those who are not (P<0.05). This highlights a statistically significant association between an increased level of deprivation (lower IMD) and the need for amputation in diabetic foot disease hence these two factors are inversely related.

## Discussion

Diabetes causes a great economic burden by direct cost on the healthcare system and indirect cost related to decreased productivity. Diabetic foot care accounts for a considerable proportion of healthcare expenditure in England. In 2014-2015, the cost of health care for ulceration and amputation is estimated at between £837 million and £962 million, accounting for approximately 1% of the NHS budget for England [9]. This is estimated to be more than the combined cost of breast, prostate and lung cancers [9]. The cost of diabetic foot care was evaluated in the UK. The approximated cost over the first year from initial presentation of healed ulcer was £2138, however it jumped to four times of that for an unhealed ulcer and eight times for an amputation secondary to an ulcer [10].

Aside from the economic strain that diabetic foot disease poses on the NHS, sufferers of this disease experience a huge impact on their quality of life, have an increased morbidity, and are at risk of premature mortality [11].

We analysed the relationship between the level of deprivation and the incidence of diabetic foot amputations in order to determine if socioeconomic deprivation was a predictor of poor outcome including amputations in this disease.

The impact of socioeconomic deprivation in diabetes as a whole is well-reported. Diabetes UK states that there is a 2.5 times greater risk of the poorest people in the UK developing diabetes compared to the national average, and a two times greater risk of these people developing diabetic complications [6]. This is confounded by the increased prevalence of risk factors including smoking, unhealthy diet, obesity, physical inactivity and poor blood pressure control amongst the more deprived areas [6].

A study by Connolly et al. [12] reported that socioeconomic status is inversely proportional to the prevalence of type 2 diabetes which possibly can be due to an increased exposure to lifestyle and environmental risk factors for type 2 diabetes mellitus [12]. Furthermore, patients living in deprived areas are significantly less likely to get high-quality diabetic care compared to the least deprived areas [13].

Diabetic foot disease is a common cause of non-traumatic limb amputation. If uncontrolled, diabetic neuropathy and peripheral arterial disease can lead to non-healing ulcers, infection and amputation [14]. These can eventually result in a decrease in quality of life [15] and an increase in disability [16], mortality [17] and economic burden [9] for the patient.

Furthermore, socioeconomic deprivation has been linked to mental health. Men living in areas of increased deprivation are 51% more likely to get major depressive disorders compared to less deprived areas [18]. In our study, the patients from more deprived areas receiving amputation were predominantly men. The mean PROMIS scores (assessing physical function, depression, pain interference and anxiety) were worse across all four domains for people living in the most socioeconomic deprived areas [19]. Furthermore, a study by Ragnarson et al. reported lower quality of life scores for patients with ulcers and major amputations compared to end-stage renal disease, prostate and breast cancer based on EQ-5D scores [20].

The five-year mortality and cost of care for diabetic foot complications are comparable to those of cancer [11]. Approximately 20% of moderate to severe diabetic foot infections result in lower extremity amputations [21]. Notably, patients with diabetic foot disease fear major amputation worse than death [22]. The five-year mortality rate for minor and major amputations is 46.2% and 56.6%, respectively [23]. In patients with coexisting chronic kidney disease and other co-morbidities the mortality rate is even higher [24].

The complications of diabetic foot disease not only affect mortality and lifespan but also greatly impact the morbidity and health span of a patient. Amputations increase the disability burden. We measure a population’s disability burden using Years Lived with Disability (YLDs). YLDs are calculated by multiplying the total number of people the condition affects by the disability weight (average condition severity). An estimated 16.8 million YLDs (2.07% of global YLDs) resulted from diabetic foot complications in 2016 [25]. Our study shows an increased prevalence of amputations in deprived areas which increases this disability burden.

Whilst our study highlights a clear trend between the level of socioeconomic deprivation and the incidence of amputations in diabetic foot disease, it is difficult to individually isolate this factor. Prospective studies on larger sample sizes with greater follow-up times could further support this hypothesis.

## Conclusions

Socioeconomic deprivation likely has a significant impact on the progression and outcome of diabetic foot disease. Our data reveals that the prevalence of amputation is four times greater in patients residing in more deprived areas compared to less deprived areas. This is comparable with deprivation and poor outcomes which is documented in a number of other diseases. As discussed, this may be confounded by variables including healthcare inequalities in areas of increased deprivation, resulting in inadequate surveillance and access to diabetic care or decreased education in these areas leading to a greater incidence of risk-taking behaviour (smoking, obesity and physical inactivity). Highlighting this issue is vital in stimulating effective change to help ensure that healthcare resources are equitably distributed to these areas of deprivation to improve outcomes and so decrease the burden on patients and the healthcare system nationally.
